# 

**Published:** 1954-10

**Authors:** 


### Contents

| | | Page |
|---|---|---|
| DAMNED DRUGS (PART II) | *Dr. G. E. F. Sutton* | 103 |
| CAT-SCRATCH DISEASE | *Dr. D. Bryant and Dr. R. P. Warin* | 113 |
| SOME NEW METHODS OF RADIOTHERAPY | *Dr. R. C. Tudway* | 116 |
| GENERAL PRACTICE | *Dr. G. F. Abercrombie* | 120 |
| COLLOID CYST OF 3rd VENTRICLE | *Clinico-Pathological Conference* | 127 |
| CORRESPONDENCE | | 130 |
| ANNOTATIONS | | 130 |
| EXTENSIONS TO THE UNIVERSITY DEPARTMENT OF MEDICINE | | |
| TOO MANY MEDICO-SOCIAL WORKERS? | | |
| NEWS FROM SOCIETIES | | 131 |
| COLLEGE OF GENERAL PRACTITIONERS | | |
| BRISTOL MEDICO-CHIRURGICAL SOCIETY | | |
| CLINICAL SOCIETY OF BATH | | |
| DEVON AND EXETER MEDICO-CHIRURGICAL SOCIETY | | |
| PLYMOUTH MEDICAL SOCIETY | | |
| SOUTH SOMERSET MEDICAL CLUB | | |
| TORQUAY AND DISTRICT MEDICAL SOCIETY | | |
| CORNWALL CLINICAL SOCIETY | | |
| B.M.A. BRISTOL DIVISION | | |
| B.M.A. GLOUCESTERSHIRE BRANCH | | |
| WEST OF ENGLAND CHILD HEALTH GROUP | | |
| NOTES AND NEWS | | 137 |
| UNIVERSITY OF BRISTOL | | |
| MEDICAL POSTGRADUATE DEPARTMENT | | |
| APPOINTMENTS | | 138 |

